# Heterochromatin and gene positioning: inside, outside, any side?

**DOI:** 10.1007/s00412-012-0389-2

**Published:** 2012-10-23

**Authors:** K. Laurence Jost, Bianca Bertulat, M. Cristina Cardoso

**Affiliations:** Cell Biology & Epigenetics, Department of Biology, Technische Universität Darmstadt, Schnittspahnstr. 10, 64287 Darmstadt, Germany

## Abstract

All cellular processes depend on the expression and repression of the right sets of genes at the right time. As each cell contains the same DNA, transcriptional and epigenetic factors have to maintain tight control over gene expression. Even a small divergence from the correct transcriptional program can lead to severe defects and even death. Having deciphered the complete linear genetic information, we need to clarify how this information is organized into the dynamic and highly heterogeneous three-dimensional space of the eukaryotic cell nucleus. Observations on the higher order organization of DNA into differentiated condensation levels date back to the early twentieth century, and potential implications of these structural features to gene expression were postulated shortly after. In particular, proximity of genes to condensed regions of heterochromatin was proposed to negatively influence their expression and, henceforward, the concept of heterochromatin as subnuclear silencing compartment emerged. Methodological advances fueled a flurry of recent studies, which only, in part, led support to this concept. In this review, we address how (hetero)chromatin structure and proximity might influence gene expression and discuss the challenges and means to unravel this fundamental biological question.

## Bits and pieces of history

**Fig. 1:**
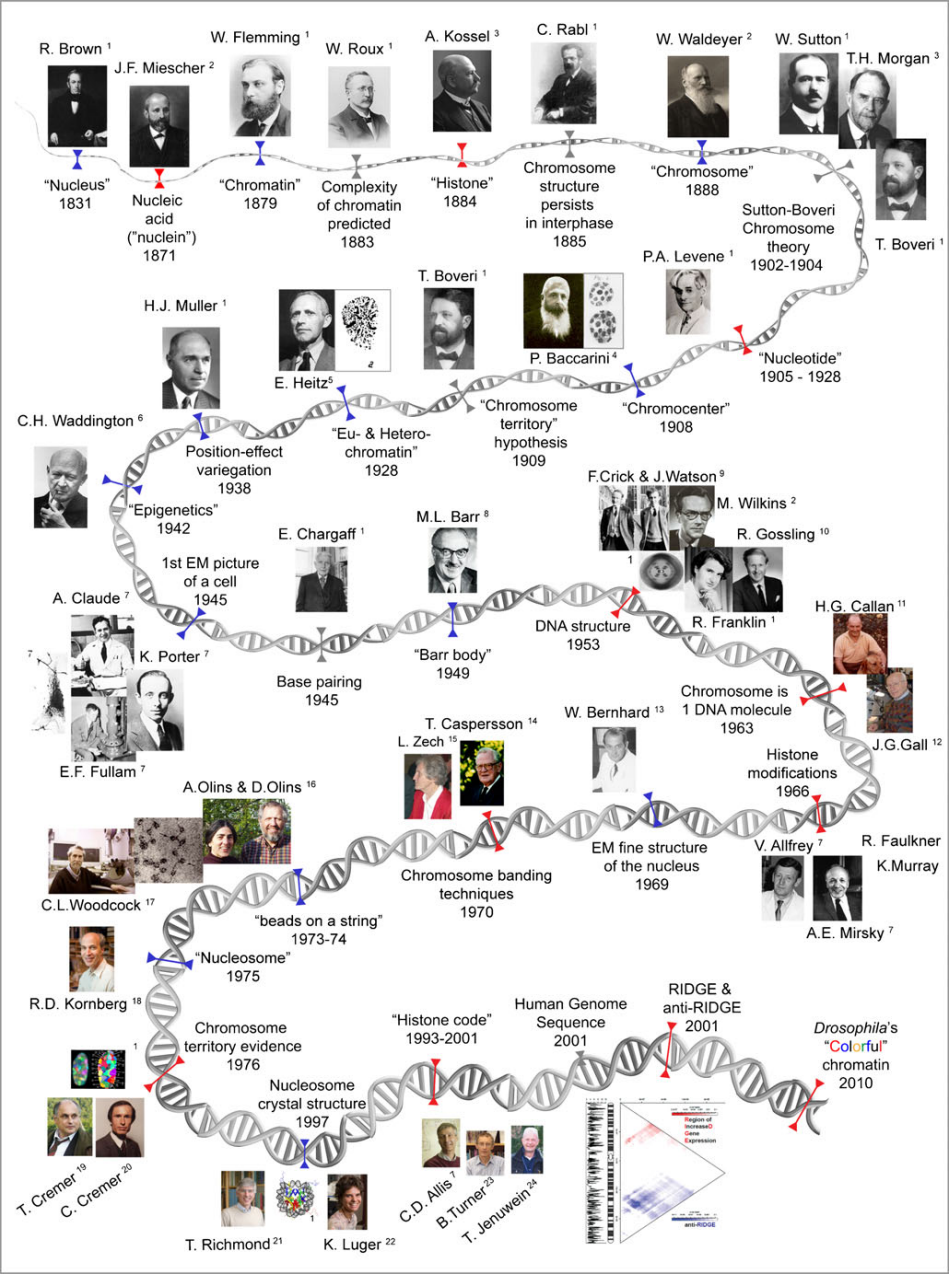
Timeline compilation of landmark discoveries and concepts (*gray*) on molecular (*red*) and cellular (*blue*) aspects of chromatin. Image sources are (1) Wikipedia; (2) MPI for the History of Science, Berlin, Germany; (3) nobelprize.org; (4) www.dipbot.unict.it/erbario_fr/Baccarini.html; (5) portrait by Esko Suomalainen in 1948; (6) http://www.che.ac.uk/what-we-do/conrad-waddington/; (7) Rockefeller University, NY, USA; (8) Canadian Medical Hall of Fame, Canada; (9) Cold Spring Harbor Laboratory Archives, NY, USA; (10) http://dnaandsocialresponsibility.blogspot.de/2010/09/dna-story-at-kings-hidden-dna-workers.html; (11) http://projects.exeter.ac.uk/lampbrush/people.htm; (12) http://hermes.mbl.edu/events/2007_events_friday/events_friday_07_27_07.html; (13) http:/wbworkshop.com/wb.html; (14) Courtesy of Prof. Niels Ringertz; (15) Deutsche Gesellschaft für Humangenetik, Germany; (16) Bowdoin College, USA; (17) UMass Amherst, USA; (18) Stanford University, Stanford Report (Photo, Linda Cicero), USA; (19) Department of Human Genetics, LMU Munich, Germany; (20) Kirchhoff Institute of Physics, University of Heidelberg, Germany; (21) ETH, Zürich, Swiss; (22) Universität Göttingen, Germany; (23) Birmingham University, UK; and (24) http://epigenome.eu/en/4,14,0

The lively history of (hetero)chromatin dates back from the nineteenth century to today’s vivid debate about its relation and influence on gene regulation (Fig. 1). Steady progress in microscopy including fixation and staining techniques led to the discovery of cells and, finally, the observation of the nucleus described first by Robert Brown in 1831 (Brown 1831). Brown’s observation immediately raised the questions: “what is the nucleus made of?” and “what is its purpose?”

Answers were found to the first question by means of chemical analysis between the 1870s and the early twentieth century. In the search for the components of the nucleus, a first success was Friedrich Miescher’s discovery of a “highly phosphorous organic acid” initially introduced as “nuclein” in 1871 (Miescher 1871) and subsequently renamed “chromatin” by Walther Flemming in 1879 (Flemming 1879), due to its affinity to aniline dyes. Further progress on the major building bricks of chromatin was made with Albrecht Kossel’s discovery of histones in 1884 (Kossel 1911). Finally, with Phoebus Levene’s work (Levene 1903; Levene and La Forge 1915) on the chemical composition of nucleotides and the identification of deoxyribose in 1928, all major chromatin components were identified (reviewed in Choudhuri 2003). Each discovery resulted in intense debates on the question: what is its purpose?

Fueled by the aftermath of Charles Darwin and Alfred R. Wallace’s evolution theory (Darwin and Wallace 1859; Darwin 1859), Wilhelm Roux predicted, already in 1883, chromatin’s complexity and importance (reviewed in Cremer and Cremer 2006). The observation of chromosomes (a term suggested by Waldeyer 1888), chromosome configuration (“Rabl configuration”), and their nonrandom distribution during cell division finally culminated in Walter Sutton (Sutton 1903) and Theodor Boveri’s (Boveri 1904) theory called as “Sutton–Boveri chromosome theory of inheritance” (reviewed in Cremer and Cremer 2006).

While chemical analysis deciphered the composition of chromatin, its organization within the cell nucleus was limited by the microscopic techniques available. However, in 1908, the Italian botanist Pasquale Baccarini reported dark stained bodies in the interphase nucleus of plants, which could be distinguished from nucleoli. Baccarini named those structures “chromocentri” or chromocenters (Baccarini 1908). Although little was known about the nature of chromocenters, they where postulated to be condensed parts of chromosomes by Rosenberg (1903). Finally, in 1928, Emil Heitz introduced the term heterochromatin and euchromatin (Heitz 1928) to describe chromosomal regions that behave differently in terms of staining and compaction throughout the cell cycle. Heitz defined heterochromatin as staining intensely and remaining compacted during the cell cycle, while euchromatin would stain lightly and disappear during telophase. He further observed a spatial relationship between heterochromatin and nucleoli. As chromocenters always stained strongly, it was rightly assumed that they consist of heterochromatin. Despite more than a century of efforts to define the structure and function of chromatin, many of the basic mechanisms that govern the formation of differentiated chromatin types remain poorly understood, and their biological role is largely unknown.

## Heterochromatin: cytological versus molecular definition

**Fig. 2:**
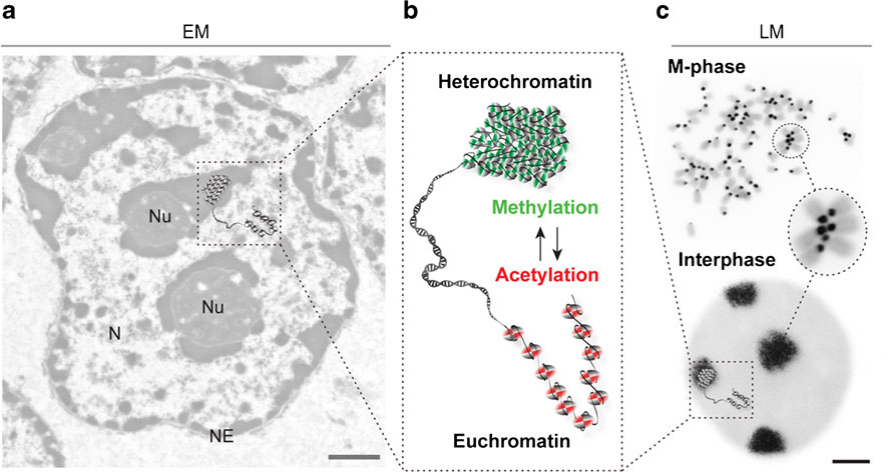
Heterochromatin: in need of definition? Historically and from a cytological point of view, Emil Heitz (see Fig. 1) distinguished hetero and euchromatin. **a** Within an exemplary electron microscopy (*EM*) picture (*left*) of a mouse liver cell nucleus (*N* nucleus, *Nu* nucleolus, *NE* nuclear envelope), heterochromatin appears as electron dense in contrast to the more open state of euchromatin. **b** With the recent advent of high-throughput epigenomics, molecular features (histone and DNA modifications) have been assigned to particular chromatin states and are shown in the simplified graphic enlarged in the center. **c** The cell cycle dynamics and cytological organization of the very condensed chromatin structures around the centromeres can be appreciated in the fluorescence light microscopy (*LM*) pictures (*right*) of M phase and interphase cells. FISH-stained mouse metaphase chromosomes and interphase cell with probes against pericentric heterochromatin (*black*) and DNA counterstaining (*gray*) are shown. Condensed pericentric heterochromatin regions from multiple chromosomes cluster together in the interphase cell nucleus forming the so-called “chromocenters.” Cytological and molecular definitions have not yet been conclusively linked together. *Scale bars* EM 0.5 μm and LM 2 μm

As described above from light microscopy observations, the cytological definition of heterochromatin is rather straightforward but also very general. This can be best appreciated in the electron micrograph image shown in Fig. 2a, where electron dense heterochromatin is visualized inside the nucleus, mostly around the nucleolus and lining the inner surface of the nuclear envelope.

From a molecular viewpoint, chromatin contains DNA and also proteins; the major components of which are the basic histone proteins (reviewed in Woodcock 2006). Histones form repeating octameric complexes called nucleosomes consisting of two of each of the core histone proteins H2A, H2B, H3, and H4 (Kornberg 1974). This constitutes the first level of chromatin compaction. Several higher order organization levels (reviewed in Woodcock and Ghosh 2010) have been proposed and give rise to increasing chromatin compaction, with the highest compaction being achieved in metaphase chromosomes (Fig. 2c). Even though the core histones are mainly the same throughout the chromatin, different histone modifications have been correlated with and, henceforth, serve as markers for different types of chromatin (reviewed in Kouzarides 2007). Of special importance are acetylation and methylation marks (Fig. 2b). The positive charge of lysine residues is neutralized through acetylation, which has been proposed to weaken the interactions between histones and DNA, leading to a more open chromatin structure, mostly correlated with the euchromatin state and gene expression. In contrast to acetylation, methylation of lysine 9 in histone H3 has been correlated with constitutive heterochromatin and gene silencing. Histone modification cannot only help to distinguish between hetero and euchromatin but also to distinguish facultative from constitutive heterochromatin, as the former is enriched in histone H3 methylated at lysine 27 (reviewed in Trojer and Reinberg 2007). With the identification of a growing number of histone modifications influencing chromatin structure, a “histone code” (Fig. 1) was hypothesized by which these posttranslational modifications could be combinatorially read by other factors and translated into different gene expression states (Strahl and Allis 2000). Potential readers of this histone code are proteins such as heterochromatin protein 1, polycomb group proteins, and many others (reviewed in Jenuwein and Allis 2001).

Methylation can, in addition, be found as a DNA modification at the position 5 of the cytosine base and is enriched in heterochromatin. How DNA methylation controls gene expression is still an open question, and direct as well as indirect mechanisms are discussed (reviewed in Leonhardt and Cardoso 2000). Methylation on the DNA level is interpreted by a family of methyl-CpG binding proteins (reviewed in Brero et al. 2006) that recruit chromatin regulators (e.g., histone deacetylases), inducing conformational chromatin changes leading to repression of gene expression (Nan et al. 1998; Zhang et al. 1999).

With the advent of high-throughput epigenomics, a catalog of molecular marks has been assigned to particular chromatin states. In this context, regions of increased gene density (RIDGEs) and opposing anti-RIDGEs (gene poor regions) were defined also, taking into consideration CpG islands, CG content, and number of short and long interspaced elements (SINEs and LINEs) (Caron et al. 2001). Recently, an association study of a set of molecular marks lead to the further discrimination of chromatin into five main types (Filion et al. 2010) (Fig. 1, “colorful chromatin”).

In spite of all the recent progress in this area, the cytological and molecular definitions of (hetero)chromatin have not yet been conclusively and comprehensively linked together. Furthermore, our understanding of the higher order architecture of chromatin and its functional consequences is far from satisfactory.

## Heterochromatin: a transcriptional silencing compartment?

One of the most important epigenetic roles of heterochromatin was recognized very early on. In 1930, Muller (1930) discovered that *Drosophila* flies treated with X-rays developed random color patterns of white and brown patches in the eyes. He could show that by random mutation, the *white* gene locus was translocated adjacent to heterochromatic regions and, thereafter, silenced. This effect was named position effect variegation (PEV). Further studies (Demerec and Slizynska 1937) broadened the knowledge about PEV, showing that genes in direct heterochromatic neighborhood were silenced before more distal genes. Altogether, these experiments showed that usually active genes get silenced just by being in the vicinity of heterochromatin and lead to the development of the concept of heterochromatin spreading. A similar effect was reported in different organisms for genes translocated to telomeric chromosomal regions and referred to as telomeric position effect variegation (TPEV) (Gehring et al. 1984; Horn and Cross 1995; Gottschling et al. 1990). (T)PEV is based on *cis* chromosomal effects, i.e., genes are affected by heterochromatin proximity within the same chromosome. Interestingly, recent work in *Caenorhabditis* indicated that large transgenic repeated arrays of tissue-specific gene promoters become heterochromatinized and gene activation within these repeats lead to looping away from the heterochromatic subnuclear domain (Meister et al. 2010). A similar looping out of heterochromatin effect upon transcription factor expression of a transgene integrated within satellite repeat-rich heterochromatin was also observed in mice (Lundgren et al. 2000). In both studies though, looping away from the heterochromatin was not always accompanied by gene activation.

In *Drosophila*, mouse, and plant cells, constitutive heterochromatin is clustered into chromocenters during interphase as depicted exemplarily in a mouse interphase cell in Fig. 2c. Chromocenters contain pericentric heterochromatin, which resides close to the centromeres and is enriched in satellite repeat sequences. Whether heterochromatin can also influence gene expression in *trans* has been more recently investigated by several groups (see below and Table 1) but remains controversial likely due to the lack of normalization standards to account for morphological and technical variability.

**Table 1:** Studies correlating gene expression and heterochromatin proximity

Heterochromatin^a^	Cells	System	No. of genes	Method	Outcome	Reference
Chromocenter	Mouse B lymphocytes	Activation of B lymphocytes	6	FISH gene colocalization to chromocenter	Correlation between position and transcription	Brown et al. (1997)
Chromocenter	Mouse B lymphocytes	Comparing B lymphocytes with transgene within or out of pericentric heterochromatin	1	Integration of transgene into chromocenters	Transgene active even inside pericentric heterochromatin	Sabbattini et al. (2001)
Chromocenter	Mouse T cells	Comparison of CD4 and CD8 (non)-expressing cells	2	FISH gene colocalization to chromocenter	Correlation between position and transcription	Delaire et al. (2004)
Chromocenter	*Drosophila* eye disk	Comparing activated versus silenced genes	3	FISH gene distance to heterochromatin	Relocation of genes away from chromocenters upon activation	Harmon and Sedat (2005)
Lamina	Myogenic mouse cells	Comparison of myogenic versus non-myogenic cells	2	FISH gene colocalization with lamina	Movement of locus away from lamina upon activation	Lee et al. (2006)
Lamina	Human mammary epithelial cells	Tumorigenesis	11	FISH and radial positioning from gene to lamina by template ellipse	For seven out of 11 genes correlation between position and transcription	Meaburn and Misteli (2008)
Lamina	Pig mesenchymal stem cells	Adipogenesis	9	FISH distance from gene to nuclei center as ratio of the nuclear radius	Correlation between position and transcription	Szczerbal et al. (2009)
Lamina	Mouse fibroblasts	Comparing expression of genes tethered or not to the lamina	Chromosomal region	Tethering of gene to lamina	Some genes remain active some get repressed	Reddy et al. (2008)
Lamina	Human HT1080 cells	Comparing expression of genes tethered or not to the lamina	Chromosomal region	Tethering of gene to lamina	Some genes remain active some get repressed	Finlan et al. (2008)
Lamina	Human U2OS cells	Comparing expression of genes tethered or not to the lamina	1	Tethering of gene to lamina	Tethered gene remained active	Kumaran and Spector (2008)
Chromocenter/lamina	Mouse neuronal precursor cells	Astrocyte differentiation	1	FISH gene colocalization to structure and radial distance to lamina	No relation to chromocenters for active or inactive loci; movement away from lamina upon activation	Takizawa et al. (2008)
Chromocenter/lamina	Mouse embryonic stem cells, induced pluripotent stem cells, and embryonic fibroblasts	Comparison of pluripotency gene localization (*Oct4*, *Sox2*, and *Nanog*) during gain/loss of pluripotency	3	FISH gene distance to heterochromatin measurement and single cell-based normalization	Internal nuclear gene positioning correlated with expression and gene relocation restricted by high gene density in neighborhood	Jost et al. (2011)

*FISH* fluorescence in situ hybridization

^a^Chromocenter corresponds to constitutive heterochromatin and lamina to facultative (peripheral) heterochromatin

**Fig. 3:**
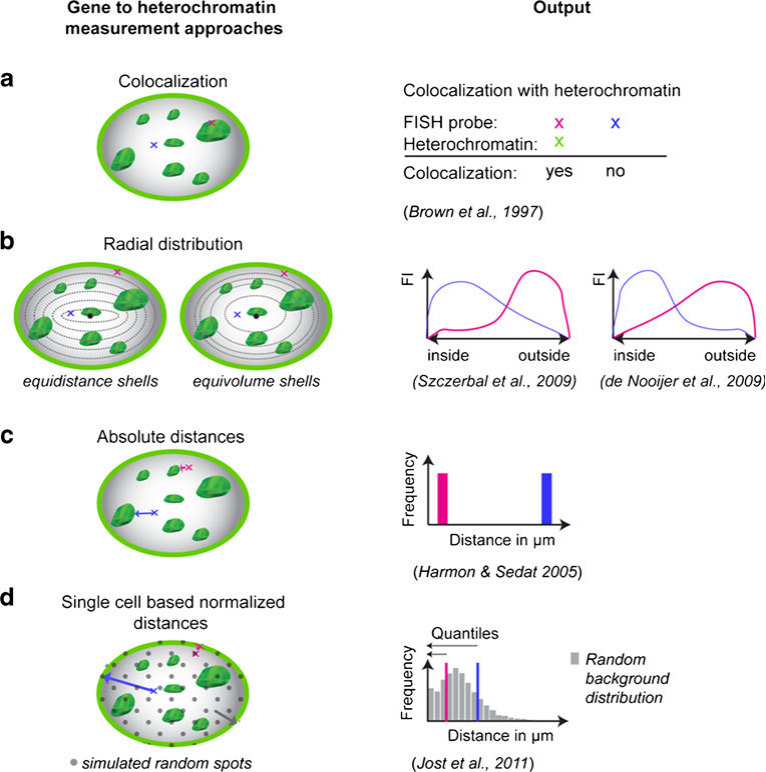
Strategies to investigate the role of heterochromatin (proximity) in gene silencing. The schemes (*left*) present alternative approaches to determine gene (*red* and *blue*) to heterochromatin (*green*) proximity in the nucleus and their respective data outcome (*right*). **a** Classic evaluation is based on simple colocalization of genes and heterochromatin yielding a clear yes or no outcome. **b** Radial distribution analysis within the cell nucleus can be performed in shells of the same distance (equidistance) or of the same volume (equivolume). Both methods yield a fluorescence intensity (*FI*) based distribution. Depending on which shell distribution is chosen, either interior or exterior genes are underestimated. **c** Comparing gene to heterochromatin absolute distances in 3D is a very unbiased method but does not take into account morphological variability. **d** The quantile-based approach combines absolute distance measurements (in 3D) with single-cell normalization (software and audiovisual tutorial available at http://www.cardoso-lab.org/publications/Randomizer.zip). Simulation and measurement of random (virtual) spots (*gray*) results in a random background distribution that normalizes for morphological differences. For each procedure and outcome, references are indicated below. An annotated list of studies is given in Table 1

Initial studies in hematopoietic cells showed a correlation between gene activity and colocalization with chromocenters (Fig. 3a). In this study, B lymphocytes were investigated, and a relocation of active genes away from chromocenters was observed, whereas silenced genes were found to colocalize with chromocenters (Brown et al. 1997). However, the number of genes studied was relatively limited, and only genes relevant to the hematopoietic system were evaluated. Follow-up studies also mainly concentrated on the hematopoietic cell system, but soon, others organisms and cell types were examined as well (Table 1). All these experiments were performed in fixed cells, thus capturing only a snapshot in time and without taking chromatin mobility into account (>2 μm in about 4 h in G1 phase cells; Walter et al. 2003). The first study that took into consideration the role of chromatin mobility was performed on *Drosophila* eye disks, comparing distances from three genes to heterochromatin in cells with active versus inactive gene loci (Harmon and Sedat 2005) (Fig. 3c). They could show a clear relation of gene silencing and proximity to heterochromatin of the same or different chromosomes. Although these studies established a correlation between gene (re)position to pericentric heterochromatin and repression, others found no support for this correlation (Sabbattini et al. 2001; Takizawa et al. 2008), suggesting that the effects might be different for different loci.

Since heterochromatin does not only accumulate in form of chromocenters but also at the periphery of mammalian nuclei, recent studies have concentrated on the nuclear periphery as a silencing compartment. This is of special interest as human cells do not exhibit clear heterochromatic clusters comparable to those found in mouse cells.

The first indication of the periphery being a transcriptional regulator was found in yeast and later in mouse myogenesis (Lee et al. 2006). In yeast, telomeres are enriched at the nuclear periphery, leading to an increased concentration of silent information regulator (Sir) proteins which cause silencing of genes (Maillet et al. 1996). Sir2 deacetylates histone tails recruiting other Sir proteins, leading to spreading of silencing (reviewed in Gasser and Cockell 2001). Silencing at the periphery through tethering of genes could also be observed for some genes albeit not for others in mouse fibroblasts (Reddy et al. 2008), mouse astrocytes (Takizawa et al. 2008), and human cancer cells (Meaburn and Misteli 2008) (see also Table 1).

Using a molecular tagging approach based on expression of DNA adenine methyltransferase (DamID) fusion to lamina components, genome-wide association studies with the nuclear lamina have been performed. Genes within lamina-associated domains (LADs) were generally found to have a lower expression level than genes outside of LADs (Peric-Hupkes et al. 2010; Guelen et al. 2008). As the DNA modification by the Dam fusion protein requires only a transient interaction with the nuclear periphery, it would be important to validate in situ the outcome of these large-scale studies.

## Heterochromatin and gene positioning: methods and challenges

The in situ heterochromatin association studies, described above, have limitations regarding their analysis (Fig. 3b). They either score association with the lamina at the nuclear periphery by simple colocalization (Lee et al. 2006) or by using radial positioning within the cell nucleus (Takizawa et al. 2008; Szczerbal et al. 2009; Meaburn and Misteli 2008). Scoring colocalization is problematic because it does not take gene mobility into account. Using a shell-based analysis (reviewed in Ronneberger et al. 2008) poses the additional problem of how the shells should be placed and whether their dimensions have biological meaning. In general, two possibilities of shell placement can be found in the literature: equidistant (Szczerbal et al. 2009) or equivolume shells (de Nooijer et al. 2009). Using one or the other can change the outcome substantially as shown on Fig. 3b. A further experimental challenge posed by the different data sets is the variability of nuclear morphology. Different cells show a great variety of shapes and sizes which is often altered upon differentiation. This challenge is acknowledged by many researchers in the field (Meaburn and Misteli 2008). Furthermore, when correlating gene positioning and heterochromatin, the distribution of heterochromatin within it also plays a role.

To circumvent this conundrum and be able to compare morphologically variable cells, we recently developed a single-cell-based normalization protocol, which compares, in each single cell, the random background distribution of distances from several thousands of simulated locations and the actual position of the gene of interest (Jost et al. 2011). The fraction of random distances smaller than the distance of the actual gene to heterochromatin is taken as the quantile value (Fig. 3d). This value is not only normalized for the specific cell size and chromatin distribution but also robust to variations of image acquisition and threshold settings. To test and validate the approach, the location of pluripotency genes in mouse stem cells, induced pluripotent stem cells, and fibroblasts was compared. The outcome did not corroborate the hypothesis that heterochromatin acts as a gene silencing compartment (Jost et al. 2011). With these new computational tools at hand, genome-wide unbiased studies should be able to settle this question. Furthermore, extending the investigations to several different in vitro and in vivo differentiation systems and organisms will be important to ascertain their general biological validity.

In summary, gene position inside or outside of heterochromatin and gene expression is not yet convincingly linked. Thus, the question persists “does a gene’s location matter?” In support of this, the evidence so far shows that genes are not randomly positioned within the nucleus, and furthermore, their location is influenced by the respective chromosomal neighborhood.

## Outlook

Even though heterochromatin is found in many species including mouse, human, and flies, and to a lower extent, even in yeast, it varies extensively in composition and architecture. The same is true for nuclear size, shape, and morphology. Both parameters (molecular composition and in situ organization) need to be integrated in future studies. Heroic attempts to perform high-throughput molecular analysis in single cells are ongoing, and the link to 3D chromatin organization might be just around the corner.

## References

[CR1] Baccarini P (1908). Sulle cinesi vegetative del “*Cynomorium coccineum* L.”. N Giorn Bot Ital N Ser.

[CR2] Boveri T (1904). Ergebnisse über die Konstitution der chromatinschen Substanz des Zellkern.

[CR3] Brero A, Leonhardt H, Cardoso MC (2006). Replication and translation of epigenetic information. Curr Top Microbiol Immunol.

[CR4] Brown R (1831). Observations on the organs and ode of fecundation in Orchideae and Asclepiadeae. Trans Linn Soc London.

[CR5] Brown KE, Guest SS, Smale ST, Hahm K, Merkenschlager M, Fisher AG (1997). Association of transcriptionally silent genes with Ikaros complexes at centromeric heterochromatin. Cell.

[CR6] Caron H, van Schaik B, van der Mee M, Baas F, Riggins G, van Sluis P, Hermus MC, van Asperen R, Boon K, Voute PA, Heisterkamp S, van Kampen A, Versteeg R (2001). The human transcriptome map: clustering of highly expressed genes in chromosomal domains. Science.

[CR7] Choudhuri S (2003). The path from nuclein to human genome: a brief history of DNA with a note on human genome sequencing and its impact on future research in biology. B Sci Technol Soc.

[CR8] Cremer T, Cremer C (2006). Rise, fall and resurrection of chromosome territories: a historical perspective. Part I. The rise of chromosome territories. Eur J Histochem.

[CR9] Darwin C (1859). On the origin of species by means of natural selection, or the preservation of favoured races in the struggle for life.

[CR10] Darwin C, Wallace AR (1859). On the tendency of varieties to depart indefinitely from the original type. J Proc Linn Soc.

[CR11] de Nooijer S, Wellink J, Mulder B, Bisseling T (2009). Non-specific interactions are sufficient to explain the position of heterochromatic chromocenters and nucleoli in interphase nuclei. Nucleic Acids Res.

[CR12] Delaire S, Huang YH, Chan SW, Robey EA (2004). Dynamic repositioning of CD4 and CD8 genes during T cell development. J Exp Med.

[CR13] Demerec M, Slizynska H (1937). Mottled White 258–18 of *Drosophila melanogaster*. Genetics.

[CR14] Filion GJ, van Bemmel JG, Braunschweig U, Talhout W, Kind J, Ward LD, Brugman W, de Castro IJ, Kerkhoven RM, Bussemaker HJ, van Steensel B (2010). Systematic protein location mapping reveals five principal chromatin types in *Drosophila* cells. Cell.

[CR15] Finlan LE, Sproul D, Thomson I, Boyle S, Kerr E, Perry P, Ylstra B, Chubb JR, Bickmore WA (2008). Recruitment to the nuclear periphery can alter expression of genes in human cells. PLoS Genet.

[CR16] Flemming W (1879). Neue Beiträge zur Kenntniss der Zelle (1st part). Arch Mikrosk Anat.

[CR17] Gasser SM, Cockell MM (2001). The molecular biology of the SIR proteins. Gene.

[CR18] Gehring WJ, Klemenz R, Weber U, Kloter U (1984). Functional analysis of the white gene of *Drosophila* by P-factor-mediated transformation. EMBO J.

[CR19] Gottschling DE, Aparicio OM, Billington BL, Zakian VA (1990). Position effect at *S*. *cerevisiae* telomeres: reversible repression of Pol II transcription. Cell.

[CR20] Guelen L, Pagie L, Brasset E, Meuleman W, Faza MB, Talhout W, Eussen BH, de Klein A, Wessels L, de Laat W, van Steensel B (2008). Domain organization of human chromosomes revealed by mapping of nuclear lamina interactions. Nature.

[CR21] Harmon B, Sedat J (2005). Cell-by-cell dissection of gene expression and chromosomal interactions reveals consequences of nuclear reorganization. PLoS Biol.

[CR22] Heitz E (1928). Das Heterochromatin der Moose.I. Jahrb Wiss Bot.

[CR23] Horn D, Cross GA (1995). A developmentally regulated position effect at a telomeric locus in *Trypanosoma brucei*. Cell.

[CR24] Jenuwein T, Allis CD (2001). Translating the histone code. Science.

[CR25] Jost KL, Haase S, Smeets D, Schrode N, Schmiedel JM, Bertulat B, Herzel H, Cremer M, Cardoso MC (2011). 3D-Image analysis platform monitoring relocation of pluripotency genes during reprogramming. Nucleic Acids Res.

[CR26] Kornberg RD (1974). Chromatin structure: a repeating unit of histones and DNA. Science.

[CR27] Kossel A (1911). Über die chemische Beschaffenheit des Zellkerns. Münch Med Wschr.

[CR28] Kouzarides T (2007). Chromatin modifications and their function. Cell.

[CR29] Kumaran RI, Spector DL (2008). A genetic locus targeted to the nuclear periphery in living cells maintains its transcriptional competence. J Cell Biol.

[CR30] Lee H, Quinn JC, Prasanth KV, Swiss VA, Economides KD, Camacho MM, Spector DL, Abate-Shen C (2006). PIAS1 confers DNA-binding specificity on the Msx1 homeoprotein. Genes Dev.

[CR31] Leonhardt H, Cardoso MC (2000). DNA methylation, nuclear structure, gene expression and cancer. J Cell Biochem Suppl.

[CR32] Levene PA (1903). On the chemistry of the chromatin substance of the nerve cell. J Med Res.

[CR33] Levene PA, La Forge FB (1915). On chondrosamine. Proc Natl Acad Sci U S A.

[CR34] Lundgren M, Chow CM, Sabbattini P, Georgiou A, Minaee S, Dillon N (2000). Transcription factor dosage affects changes in higher order chromatin structure associated with activation of a heterochromatic gene. Cell.

[CR35] Maillet L, Boscheron C, Gotta M, Marcand S, Gilson E, Gasser SM (1996). Evidence for silencing compartments within the yeast nucleus: a role for telomere proximity and Sir protein concentration in silencer-mediated repression. Genes Dev.

[CR36] Meaburn KJ, Misteli T (2008). Locus-specific and activity-independent gene repositioning during early tumorigenesis. J Cell Biol.

[CR37] Meister P, Towbin BD, Pike BL, Ponti A, Gasser SM (2010). The spatial dynamics of tissue-specific promoters during *C*. *elegans* development. Genes Dev.

[CR38] Miescher F (1871). Über die chemische Zusammensetzung der Eiterzellen. Hoppe-Seyler’s Med-Chem Unt.

[CR39] Muller H (1930). Types of visible variations induced by X-rays in *Drosophila*. J Genet.

[CR40] Nan X, Ng HH, Johnson CA, Laherty CD, Turner BM, Eisenman RN, Bird A (1998). Transcriptional repression by the methyl-CpG-binding protein MeCP2 involves a histone deacetylase complex. Nature.

[CR41] Peric-Hupkes D, Meuleman W, Pagie L, Bruggeman SW, Solovei I, Brugman W, Graf S, Flicek P, Kerkhoven RM, van Lohuizen M, Reinders M, Wessels L, van Steensel B (2010). Molecular maps of the reorganization of genome-nuclear lamina interactions during differentiation. Mol Cell.

[CR42] Reddy KL, Zullo JM, Bertolino E, Singh H (2008). Transcriptional repression mediated by repositioning of genes to the nuclear lamina. Nature.

[CR43] Ronneberger O, Baddeley D, Scheipl F, Verveer PJ, Burkhardt H, Cremer C, Fahrmeir L, Cremer T, Joffe B (2008). Spatial quantitative analysis of fluorescently labeled nuclear structures: problems, methods, pitfalls. Chromosom Res.

[CR44] Rosenberg, O (1903) Das Verhalten der Chromosomen in einer hybriden Pflanze. Ber. d. deutschen, bot. Gesellschaft. Bd. 21

[CR45] Sabbattini P, Lundgren M, Georgiou A, Chow C, Warnes G, Dillon N (2001). Binding of Ikaros to the lambda5 promoter silences transcription through a mechanism that does not require heterochromatin formation. EMBO J.

[CR46] Strahl BD, Allis CD (2000). The language of covalent histone modifications. Nature.

[CR47] Sutton WS (1903). The chromosomes in heredity. Biol Bull Mar Biol Lab Woods Hole.

[CR48] Szczerbal I, Foster HA, Bridger JM (2009). The spatial repositioning of adipogenesis genes is correlated with their expression status in a porcine mesenchymal stem cell adipogenesis model system. Chromosoma.

[CR49] Takizawa T, Meaburn KJ, Misteli T (2008). The meaning of gene positioning. Cell.

[CR50] Trojer P, Reinberg D (2007). Facultative heterochromatin: is there a distinctive molecular signature?. Mol Cell.

[CR51] Waldeyer W (1888). Über Karyogenese und ihre Beziehung zu den Befruchtungsvorgängen. Arch Mikrosk Anat.

[CR52] Walter J, Schermelleh L, Cremer M, Tashiro S, Cremer T (2003). Chromosome order in HeLa cells changes during mitosis and early G1, but is stably maintained during subsequent interphase stages. J Cell Biol.

[CR53] Woodcock CL (2006). Chromatin architecture. Curr Opin Struct Biol.

[CR54] Woodcock CL, Ghosh RP (2010). Chromatin higher-order structure and dynamics. Cold Spring Harb Perspect Biol.

[CR55] Zhang Y, Ng HH, Erdjument-Bromage H, Tempst P, Bird A, Reinberg D (1999). Analysis of the NuRD subunits reveals a histone deacetylase core complex and a connection with DNA methylation. Genes Dev.

